# A microcomputer data-acquisition and cumulative reporting system for oestrogen and creatinine continuous-flow analysis

**DOI:** 10.1155/S1463924683000061

**Published:** 1983

**Authors:** D. Keating, G. J. Dunlop, A. L. Evans, R. A. Gowdie, N. L. Gregory, T. Lee, L. G. S. Rao

**Affiliations:** 1 West of Scotland Health Boards Department of Clinical Physics & Bio-Engineering 11 West Graham Street Glasgow G4 9LF UK; 2 Department of Biochemistry Bellshill Maternity Hospital Lanarkshire ML4 3JV UK


Journal of Automatic Chemistry, Volume 5, Number 1 (January–March 1983), pages 14–17

### A microcomputer data-acquisition and cumulative reporting system for oestrogen and creatinine continuous-flow analysis

**D. Keating, G. J. Dunlop, A. L. Evans, R. A. Gowdie, N. L. Gregory, T. Lee**

*West of Scotland Health Boards, Department of Clinical Physics & Bio-Engineering, 11 West Graham Street, Glasgow G4 9LF, UK*

**and L. G. S. Rao**

*Department of Biochemistry, Bellshill Maternity Hospital, Lanarkshire ML4 3JV, UK*

### Introduction

It has been shown that foetal death can be predicted by measuring oestrogen/creatinine ratios on early morning samples of urine and monitoring the trend in results over the last three months of a pregnancy [1].

Bellshill Maternity Hospital serves the Lanarkshire area, which has a birth rate of 10 000 per year. The urinary oestrogen/creatinine ratio test provides inexpensive antenatal monitoring for all patients in the area. The analysis, calculations and cumulative reporting of a test on this scale is both tedious and time-consuming. The introduction of a microcomputer-based system has allowed the full use of this service in Lanarkshire and oestrogen/creatinine analysis can now be carried out on up to 1000 patient samples per week. Only one technician is required for operation and data acquisition, calculations and printing of cumulative results with graphs are performed by the microcomputer system. Disc storage is available for up to 2000 patients.

No commercial instrument is yet available to perform and automate these tests.

### System design

A block diagram of the complete system is shown in figure 1. It can conveniently be described as a data-acquisition system (DAS) linked to a data-processing system.

### *Data acquisition*

The analogue signals for the DAS are obtained from retransmitting outputs of the chart-recorder of the analytical system. The DAS has no effect on the existing analyser; so, if necessary, the original method of obtaining results from the chart may still be used. The chart-recorder still functions as a useful monitoring instrument for the quality of the peak data and can be used by the operator to detect chemistry problems, such as short sampling or excess carry-over.

The analogue signals from the analyser are in the form of two asynchronous channels, each consisting of a series of peaks and troughs corresponding to each sample. The timing of the DAS is initiated by the first two peaks in each channel which are standards, subsequent peaks are measured and stored in a buffer. The DAS assumes linear calibration and performs digitization to 8-bit accuracy every 2·6 s. If a peak boundary is not detected within the predicted time interval, a missed-peak error flag is set. Another error flag is used to indicate off-scale peaks.

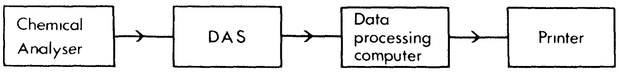

*Figure 1. Principal components of the complete system.*

The DAS has sufficient storage for three full trays (150 samples) of peak data and is able to handshake with the data-processing microcomputer. Therefore, the DAS requires a low priority from the data-processing microcomputer and no results are lost if the second microcomputer cannot be interrupted, for example, during disc access. The modular design of the DAS allows for expansion to eight channels. The DAS can output peak results directly to a printer if desired; this is useful as a checking facility and if the data-processing system is not operational.

Three levels of self-test have been incorporated. Combinations of hardware and software allow the operator or service engineer to see the current status of the system, to test the basic operation of the data-acquisition module and to monitor the internal timers and data stores during normal execution of the program.

When the unit is switched on or reset, test routines check the essential subunits, the RAM, ROM and the input-output ports. Defects in any of these will illuminate an indicator light-emitting diode (LED) on the front panel.

While the system is running normally, its status is indicated by further LEDs. One of these is held off by the program: it lights only in the case of a complete program 'crash'. Another flashes at each digitization, a third indicates the detection of a peak, and a fourth indicates when data is available for output.

### *Data processing*

Patient details are typed in from the VDU by the operator during the run. They can be printed-out as a 'work-sheet'. The data-processing microcomputer performs the calculation at the end of the run and the work-sheet can then be printed-out again with the results filled in.

The microcomputer has two disc drives using double-sided discs, the capacity of each is 1 Mbyte. Using one disc for programs and the other for data, records for up to 2000 patients are stored on disc.

### Description of the system

### *Hardware*

Both microcomputers are S100-bus-based systems. The S100 bus was chosen because it encouraged a modular design and because a wide range of commercially available boards is available thus reducing development time.

*Data-acquisition microcomputer*

The boards used in the DAS are:

(1) 8080 cpu with 'Jump on Reset' facility.
(2) 16 k RAM.
(3) 1 ROM card with four 1 k × 8 EPROMS, two for system programs, one for routine test and one for diagnostics.
(4) Serial and parallel interface card. Two serial ports send results to the data-processing microcomputer and printer. A parallel port is used for handshaking during these transfers.
(5) ADC/DAC card with eight channels of multiplexed analogue input and two analogue-output channels.
(6) Real-time clock and vector-interrupt controller card.
(7) A card providing balanced amplification between the chart-recorder and the ADC.
(8) A card controlling the front-panel status lights.

*Data-processing microcomputer*

The microcomputer runs under the CP/M operating system with 64 k of memory.

The hardware used is:

(1) Parallel/serial I/O card (for printer and VDU).
(2) Serial I/O card (interfacing to DAS microcomputer).
(3) Z80 cpu.
(4) 2 × 32 k RAM cards.
(5) Disc controller card.
(6) 2 × Y.E. data disc drives.

Other peripherals include a line-printer and VDU.

### *Software*

*Data acquisition*

The DAS system programs are written in 8080 assembly language and are organized in two major segments—the main program and the interrupt segment (figure 2).

The main program starts by initializing the system, it sets up flags, address pointers and timers and then waits in a loop until data has been placed in the output buffers. The interrupt routine supplies the data to the output buffers, incrementing last-data-entered pointers and the printer pointer, and if a difference exists between them calls the output routine. The output routine includes a code-conversion routine for translating to decimal numbers and for printing warning characters when error-flag bits are set.

*Data processing*

All programs in the data-processing microcomputer are written in Basic. The programs are all compiled, increasing the speed by a factor of 10 over interpreter versions.

The interrupt routine was written as an assembly language program which is poked into memory by Basic. This routine incorporates parity checking on the transmitted data and is placed in a protected area of memory. When the DAS wishes to send data to the data-processing microcomputer it creates an interrupt. A burst of digitized data is transferred from the DAS

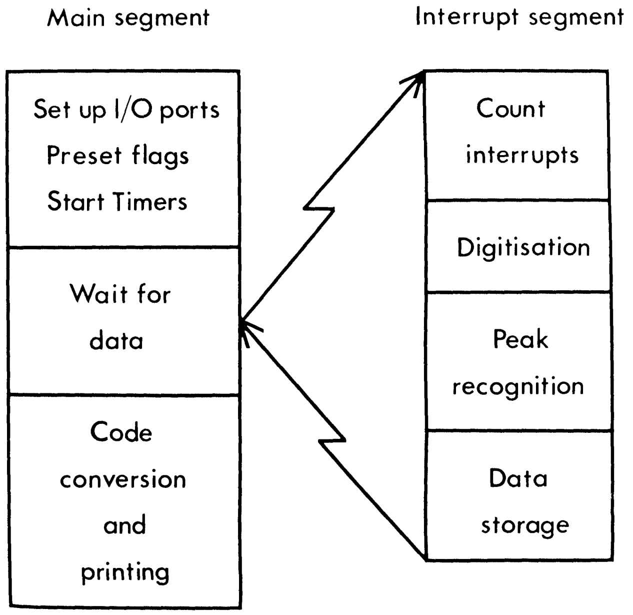

*Figure 2. DAS program structure: timer generated interrupts are used for data acquisition, completed results are sent to the data-processing microcomputer as soon as they are ready.*

to the protected area of memory in the data-processing microcomputer. The data is then transferred to peak and base-line areas of memory, leaving the buffer area free for the next line of data.

The drive A disc is the program disc and drive B disc contains the main patient file of 2000 patient records. Each patient record is 128 bytes long—holding patient details and up to 25 results (figure 3).

Only the 25 most recent results are retained on file. An index file is also stored on this disc, capable of holding 2000 patient numbers. Each index record is 120 bytes in length and can store 20 six-digit patient numbers. Record number 101 is reserved for a 'next record indicator'. A tray file on the drive A disc has the same format as the main patient file, with up to 50 patient records.

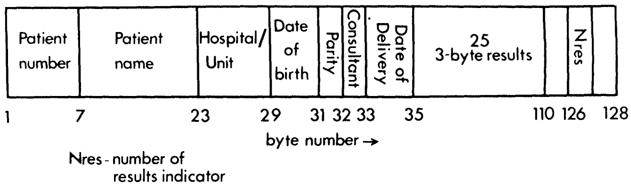

*Figure 3. Patient record structure.*

*Operating procedure*

The main Basic program displays a menu from which all others are run (see table 1). The operator can run the options in any order; a common procedure is shown in figure 4.

*Start new tray*

Entering option 4 runs the program for clearing the protected area of memory.

*Table 1. Option table.*

1 ALTER SAMPLE NO LIMIT
2 DISPLAY DATA ACQUIRED
3 CALCULATE AND PRINT RESULTS
4 START NEW TRAY
5 ALTER SAMPLES
6 DISPLAY AND PRINT WORK SHEET
7 CLOSE DOWN
8 ENTER PATIENT RECORDS
9 UPDATE MAIN PATIENT FILE
10 PRINT GRAPHS FROM TRAY FILE
11 PRINT GRAPHS FROM MAIN PATIENT FILE

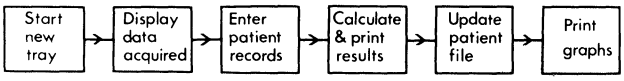

*Figure 4. Option flow diagram.*

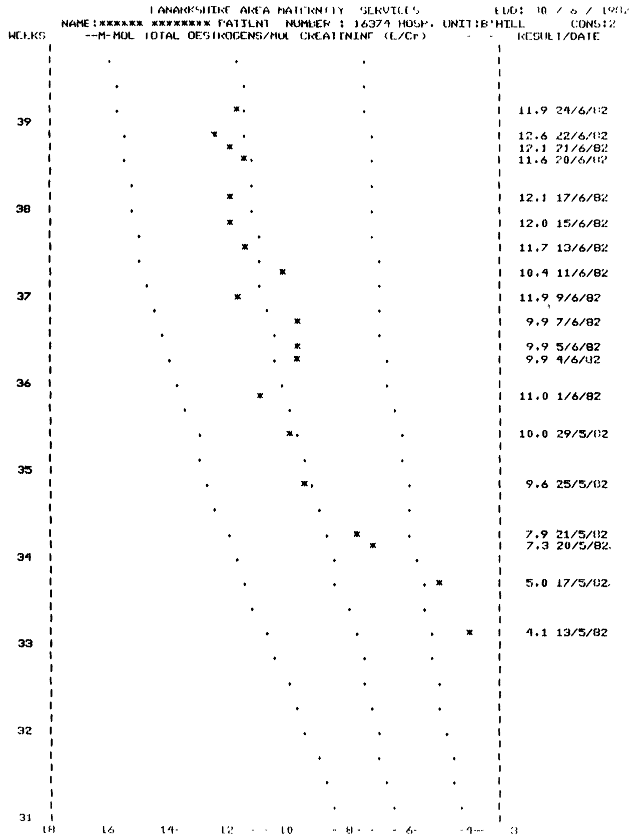

*Figure 5. Example of output: patient results are plotted as asterisks, dots are used to plot mean, 5th and 95 centile lines.*

*Display data acquired*

Option 2 displays each line of data as it is transferred from the DAS to the data-processing microcomputer.

*Enter patient records*

Option 8 enables the operator to enter patient details for each patient sample. This program is responsible for the file handling and data storage of patient records.

The patient numbers already stored on disc are read into an array in memory from the master index file. When the operator types in a patient number the program searches through the array to see of the patient is already on file. If the patient number is found, the relevant record is read from the main patient file and copied into the tray file. Patient details are displayed on the VDU to ensure reliable patient identification. If the patient is not already on file the program branches to the routine for entering patient details. The new patient record is stored in the tray file and the main patient file. The index file is updated. The index system is used because it is much faster in searching through an array in memory and then searching a disc.

When all patient details have been entered a tray file of records, including past results, exists on the drive A disc.

*Calculate and print-out results*

The option 3 program reads the digitized heights from memory, calculates concentration values for oestrogen and creatinine, and stores the ratios in an array in memory. The results are printed-out in tabular form. New results are added into the tray file at the appropriate file position.

*Update main patient file*

Option 9 copies the data from the tray file into the main patient file, effectively updating the patient records with the new results.

*Print graphs (options 10 and 11)*

A simple graph (figure 5) is plotted on the line-printer either for a tray of results or for individual patients. Graphs can be plotted by entering a tray number, a patient number or an index record number. A table of cumulative results is also produced.

*Close-down*

Option 7 is run at the end of a day. Cumulative results in tabular form are printed on the line-printer. The results are printed in alphabetical order for all patients whose samples have been analysed on that particular day; this alphabetical listing has been found to provide a useful back-up facility in the event of a microcomputer breakdown.

*Other options*

*Alter sample number limit (option 1):* allows the operator to change the sample number limit from the default value of 42 to any desired value.

*Alter samples (option 5):* this program allows manual alteration of results.

*Display and print work-sheet (option 6):* a work-sheet of patient names and patient numbers is printed on the line-printer.

Data is still transmitted from the DAS to the data-processing microcomputer while the operator is running any of the options from the menu. When there is a disc access, the data-processing microcomputer disables interrupts thus stopping the DAS from sending data.

### Discussion

In this biochemical test the parameter required is the ratio of oestrogen to creatinine in each sample. Correct synchronization of the two analysis channels is essential. This synchronization has been maintained without difficulty.

In order to establish that the 'digital' system was introducing no systematic errors, and also to assess the precision of the results achieved, the DAS was run for some time in parallel to the previous 'manual' system of peak measurement. Analysis of trays of samples from the normal clinical work-load were repeated, giving four sets of results for each tray: two for the manual method, and two for the digital method. From these results, three variances were extracted: one for the manual method of peak measurement, one for the digital method of peak measurement, and one from the process of peak production, the analytical method itself. Typical results showed the manual and digital methods to be of the same order of variance, with coefficients of variation of the order of 0·4%. As this was better, by a factor of three, than typical estimates of the coefficients of variation of the 'chemistry', the digital method was considered to be performing adequately. Its operational advantages over the manual method are those of speed and the elimination of fatigue, which leads to an increased work-load, and also the elimination of the occasional human errors which occur in manually processed data.

The total cost of the complete system, including such peripherals as the VDU and the printer, was £6000. The authors would be pleased to provide details of both the software and hardware on request.

Although the high reliability and modest development cost have vindicated the original choice of S100-bus-based systems, the advent of single-board microcomputers now offers an attractive alternative for the data-acquisition part of the system.

The data-acquisition system has been in operation since November 1979 and the cumulative reporting system since May 1982; these now provide a comprehensive reporting system for the Lanarkshire area.

### Reference

1. Rao, L. G. S., *British Medical Journal*, **2** (1977), 874.

### CONFERENCE ANNOUNCEMENT

*Analyticon 83: 12 to 14 October 1983*

Organized by SIMA—the Scientific Instrument Manufacturers' Association of Great Britain—Analyticon 83 will coincide with the Laboratory 83 exhibition. Both events will be held at the Barbican Centre in London.

Analyticon's conference programme will be divided into six main themes: organic structure, chromatography, surface analysis, elemental analysis, computers in analytical chemistry, and clinical analysis. Speakers include Professor T. S. West (Macaulay Institute, Aberdeen), Professor D. Betteridge (University College, Swansea), Dr Deans (ICI, Wilton), Professor Brooks (Glasgow University), Dr F. L. Mitchell (Clinical Research Centre, Harrow) and Dr V. E. Cosslet (Cavendish Laboratory, Cambridge).

*Further information from Mr G. C. Young, SIMA, Leicester House, 8 Leicester Street, London WC2H 7BN.*

### NOTES FOR AUTHORS

*Journal of Automatic Chemistry* covers all aspects of automation and mechanization in analytical, clinical and industrial environments. The *Journal* publishes original research papers; short communications on innovations, techniques and instrumentation, or current research in progress; reports on recent commercial developments; and meeting reports, book reviews and information on forthcoming events. All research papers are refereed.

### Manuscripts

Two copies of articles should be submitted to the Editor. All articles should be typed in double spacing with ample margins, on one side of the paper only. The following items should be sent: (1) a title-page including a brief and informative title, avoiding the word 'new' and its synonyms; a full list of authors with their affiliations and full addresses; (2) an abstract of about 250 words—this should succinctly describe the scope of the contribution and highlight significant findings or innovations; it should be written in a style which can easily be translated into French and German; (3) the main text with sections and subsections numbered; (4) appendices (if any); (5) references; (6) tables, each table on a separate sheet and accompanied by a caption; (7) illustrations (diagrams, drawings and photographs) numbered in a single sequence from 1 upwards and with the author's name on the back of every illustration; captions to illustrations should be typed on a separate sheet.

### References

References should be indicated in the text by numbers following the author's name, i.e. Skeggs [6]. In the reference section they should be arranged thus:

to a journal

Manka, D. P., *Journal of Automatic Chemistry*, **3** (1981), 119.

to a book

Malmstadt, H. V., in *Topics in Automatic Chemistry*, Ed. Stockwell, P. B. and Foreman, J. K. (Horwood, Chichester, 1978), p. 68.

### Illustrations

Line diagrams are preferred to photographs. Original copies of diagrams and drawings should be supplied, and should be drawn to be suitable for reduction to the page or column width of the *Journal*, i.e. to 85 mm or 179 mm, with special attention to lettering size. Photographs may be sent as glossy prints or as negatives.

### Proofs and offprints

The principal or corresponding author will be sent galley proofs for checking and will receive 50 offprints free of charge. Additional offprints may be ordered on a form which accompanies the proofs.

*Manuscripts should be sent to the Editor: Dr Peter B. Stockwell, Plasma-Therm Ltd, Unit 3, 2/3 Kangley Bridge Road, Lower Sydenham, London SE26 5AR.*

